# Supplementary material for: Bayesian refinement of protein structures and ensembles against SAXS data using molecular dynamics
Source: PLoS Comput Biol. 2017 Oct 18;13(10):e1005800. doi: 10.1371/journal.pcbi.1005800 (PMC5662244; doi:10.1371/journal.pcbi.1005800)
Supplement: S1 Archive — (BZ2) [file pcbi.1005800.s011.tar.bz2 › gromacs_4.62_bayesian_saxs_refi/share/html/online/gmxdump.html]

gmxdump


| gmxdump Main Table of Contents |

**VERSION 4.6  
Sat 19 Jan 2013**

---

### Description

gmxdump reads a run input file (.tpa/.tpr/.tpb),
a trajectory (.trj/.trr/.xtc), an energy
file (.ene/.edr), or a checkpoint file (.cpt)
and prints that to standard output in a readable format.
This program is essential for checking your run input file in case of
problems.

The program can also preprocess a topology to help finding problems.
Note that currently setting GMXLIB is the only way to customize
directories used for searching include files.

### Files

| option | filename | type | description |
| --- | --- | --- | --- |
| **-s** | topol.tpr | Input, Opt. | Run input file: tpr tpb tpa |
| **-f** | traj.xtc | Input, Opt. | Trajectory: xtc trr trj gro g96 pdb cpt |
| **-e** | ener.edr | Input, Opt. | Energy file |
| **-cp** | state.cpt | Input, Opt. | Checkpoint file |
| **-p** | topol.top | Input, Opt. | Topology file |
| **-mtx** | hessian.mtx | Input, Opt. | Hessian matrix |
| **-om** | grompp.mdp | Output, Opt. | grompp input file with MD parameters |

### Other options

| option | type | default | description |
| --- | --- | --- | --- |
| **-[no]h** | bool | no | Print help info and quit || **-[no]version** | bool | no | Print version info and quit || **-nice** | int | 0 | Set the nicelevel || **-[no]nr** | bool | yes | Show index numbers in output (leaving them out makes comparison easier, but creates a useless topology) || **-[no]sys** | bool | no | List the atoms and bonded interactions for the whole system instead of for each molecule type |

### Known problems

- Position restraint output from -sys -s is broken

---

http://www.gromacs.org  
gromacs@gromacs.org
